# Analysis of Torque Maintenance and Fracture Resistance after Fatigue in Retention Screws Made of Different Metals for Screw-Retained Implant-Borne Prosthesis Joints

**DOI:** 10.1155/2021/9693239

**Published:** 2021-11-18

**Authors:** Maria Beatriz Bello Taborda, Gabriela Sumie Yaguinuma Gonçalves, Cecília Alves de Sousa, Wirley Gonçalves Assunção

**Affiliations:** ^1^Department of Dental Materials and Prosthodontic, Sao Paulo State University (UNESP), Araçatuba, Brazil; ^2^Department of Dental Materials and Prosthodontic, São Paulo State University (UNESP), Araçatuba School of Dentistry, São Paulo, Brazil

## Abstract

**Purpose:**

The aim was to evaluate the effect of different metallic alloys used in the manufacture of retention screws for universal cast to long abutment (UCLA) abutments for external hexagon (HE) and Morse taper (MT) connection implants, as well as of mechanical cycling on torque maintenance and fracture resistance through electromechanical fatigue testing by mastication followed by compression testing.

**Methods:**

Sixty implants were used, 30 MT and 30 HE, with their respective titanium UCLA abutments and retention screws of 5 different materials (*n* = 6): Ti cp grade 2, Ti cp grade 4, Ti cp grade 4 hard, Ti grade 5—Ti6Al4V and surgical steel (DSP® Biomedical). The assemblies were positioned in an electromechanical masticatory fatigue testing machine. The fracture strength test was performed by compression testing in a universal testing machine EMICDL-200.

**Results:**

The cycled screws and new screws of each alloy group for each connection type were evaluated, obtaining the maximum force (FM), in order to verify the effect of mechanical cycling. The data were tabulated and submitted to appropriate statistical analysis (*α* = 0.05).

**Conclusion:**

It was concluded that for the MT, the alloy with the best performance was steel, both in the maintenance of torque and in the compression test, and cycling negatively influenced the maintenance of preload for this connection. The alloy material did not influence torque maintenance for HE. The new screws that were subjected to EMIC showed higher strength. The alloy with the lowest strength was Ti grade 2.

## 1. Introduction

Despite being a consolidated technique [1], implant-supported prostheses are still susceptible to biomechanical failures and complications [2, 3], more specifically, those that interfere with the stability of the interface region between the implant, abutment, cylinder, and retention screw. As a result, among several complications, loosening of the retention screw may occur, predisposing it to fracture [2, 4, 5].

The connection between the prosthetic pillar and the implant by means of a retention screw is called screwed connection, and the tightening force exerted on the screw is called torque [6, 7]. Once applied, it develops a compressive force between the parts that holds the components together, called preload [7]. During the masticatory cycle, there is an incidence of loads on the prosthetic structure, and these external forces, when higher than the preload value, can lead to loosening or even fracture of the screw [8, 9].

Analyzing from a mechanical-prosthetic point of view, the main reasons for the fracture of a retention screw are misadjusted infrastructure, occlusal overload, and parafunction [10, 11]. Therefore, retaining screws are prone to fracture, as it usually occurs after the screw joint is subjected to a long period of stress under multidirectional forces that exceed the preload value, such as premature lateral excursive contacts, too tight interproximal contacts, nonpassive fit of restorations, and parafunctional habits [12, 13] leading to joint separation, suggesting that a small defect will progressively reach a critical size and cause fracture [14]. Thus, screw loosening and fracture may represent a warning, indicating that too much force and load are being applied to the prosthesis [15, 16].

The need to prevent fractures of the retention screw in the screw joint resulted in modifications in properties regarding its design, composition, stem length, body, diameter, number of threads, and different values in the application of torque [17]. In general, it was necessary to evolve the characteristics of the retaining screw to maximize preload and minimize the loss of input torque during thread friction, on the assumption that increasing the stem length helps to achieve optimal elongation and shorter thread lengths reduce friction [12, 18, 19]. Moreover, the most significant factor that gives the screw good qualities is its composition, since the frictional resistance between the implant internal threads and the screw threads, composed of two different metals, can limit the preload leading to fracture [12].

It is essential that the material that composes the retaining screw has properties that, in addition to providing adequate resistance to fracture, favor preload maintenance and support the forces generated by the screw [20]. Most retention screws are made of titanium and its alloys. In a study by Assunção et al. [20], in which a comparison was made between titanium alloy screws (Ti grade 5—Ti6Al4V), gold screws, Ti grade 5 (Ti6Al4V) with diamond surface, and Ti grade 5 (Ti6Al4V) screws with surface treated with aluminum nitride blasting, the group of titanium screws (Ti grade 5—Ti6Al4V) showed the highest value of torque maintenance (81.42% ± 3.57%).

Titanium alloys are widely used when it comes to implants and their components; however, they present mechanical properties not always favorable. Pure titanium (Ti grade 2 and Ti grade 4), for example, has the disadvantage of low potential mechanical strength and low wear resistance [21], eventhough it is the main material used in implant dentistry. It is the main material used in implant dentistry. Ti6Al4V (Ti grade 5), on the other hand, has good mechanical properties, but may cause toxic effects to patients due to the vanadium and aluminum present in its composition [22]. Grade 4 hard Ti, an alloy modified by a severe plastic deformation method [23], is considered more resistant to compression and fatigue than those conventionally manufactured [24]. As for stainless surgical steel, its main disadvantage is possible corrosion, which is why it is generally used in temporary implants and, nowadays, in mini-implants in orthodontics, as it is biocompatible [25] and has good mechanical properties [26, 27], as well as being resistant, reducing the risk of fracture [28, 29].

Thus, it is valuable for clinicians to understand the mechanical characteristics of materials and the biomechanics of preload and determine the importance of variables that may interfere in this process before applying any external load [30] to achieve a stable abutment-implant junction [31]. Therefore, this study aimed to analyze the complications mentioned above, relating the torque maintenance with the fracture resistance of universal cast to long abutment (UCLA) abutment retention screws made of 4 titanium alloys (Ti cp grade 2, Ti cp grade 4, Ti cp grade 4 hard, and Ti grade 5—Ti6Al4V) and surgical steel for external hexagonal connection and Morse taper implants, submitted to mechanical cycling.

## 2. Objectives and Hypothesis

This research evaluated the effect of 5 different types of metals used in the manufacture of retention screws (Ti cp grade 2, Ti cp grade 4, Ti cp grade 4 hard, Ti grade 5—Ti6Al4V, and surgical steel) of UCLA abutments for external hexagon and Morse taper implants on torque maintenance, before and after electromechanical fatigue testing by mastication, as well as their fracture resistance by compression testing.

The null hypothesis of this study was that the different materials analyzed, the mechanical cycling, and the type of connection would not influence the torque maintenance and the fracture resistance of the retention screws in the different connections.

## 3. Materials and Methods

### 3.1. Formation of Study Groups

Our study is an in vitro study; it was not used in humans or animals; therefore, it does not fit the EQUATOR guidelines.

Sixty implants were used, 30 with Morse taper connections (MT) and 4.0 mm platform and 30 with external hexagon (HE) and 4.1 mm platform and their respective titanium UCLA abutments and retention screws. The screws were made of five different tested materials (*n* = 6), being Ti cp grade 2, Ti cp grade 4, Ti cp grade 4 hard, Ti grade 5—Ti6Al4V, and surgical steel (DSP® Biomedical, Campo Largo, Paraná, Brazil).

**Figure 1:**
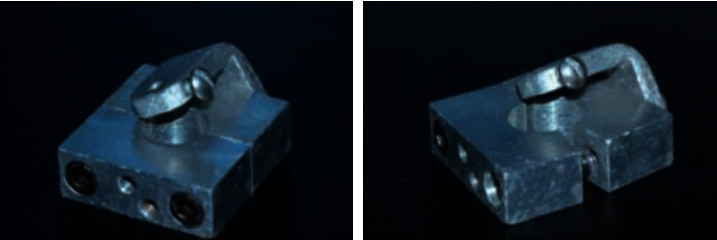
Bipartite metallic matrix for embedding of osseointegrated implants.

A bipartite metal matrix (Figure 1) was used to position the analogs, allowing a 30° inclination in relation to the vertical axis (Standard ISSO 14801-2016) [32]. A polyurethane (Polyurethane F160, Axson Brazil, São Paulo, Brazil) was used to embed the implants, as it has uniform elastic properties and a modulus of elasticity close to that of human bone tissue [33]. Once embedded, the implants received the UCLA metal prosthetic abutments, and the retention screw referring to each group and over them a hemispherical device (metal cap), on which loading was given, ensure the application of the load in the longitudinal axis, according to the Technical Standard ISO 14801 of 2016.

### 3.2. Torque Application and Measurement of the Remaining Torque (Destorque)

**Figure 2:**
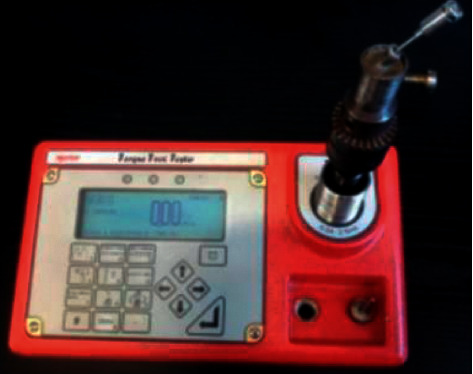
Digital torque gauge (torque tool tester, TST série 2 Norbar®, Navi Mumbai, India).

A digital torque meter (torque tool tester, TST series 2Norbar®, Navi Mumbai, India) was used for torque application and assessment of untorque (Figure 2), following a sequence previously established by a randomization process. The torque was applied according to the manufacturer's recommendations, and after a time interval of three minutes, the torque loss was measured [34, 35]. At this reading, considered as initial torque destorque, the remaining torque before mechanical cycling was evaluated. Then, the screws received another torque, called confirmation torque, to be subjected to electromechanical testing of fatigue by mastication, and then, the final torque measurements were taken (postcycling).

### 3.3. Electromechanical Chewing Fatigue Test

**Figure 3:**
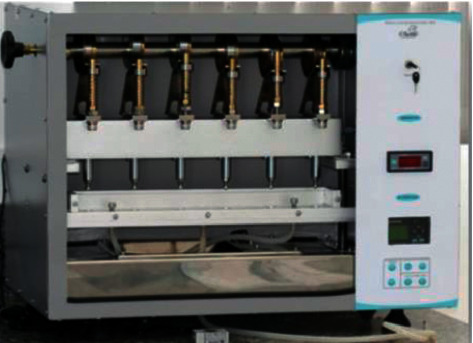
Electromechanical mastication fatigue testing equipment.

The specimens (implant/abutment/retention screw) were positioned in an electromechanical testing machine of fatigue by mastication (MSFM–ELQUIP, Equipamentos para Pesquisa Odontológica, São Carlos, SP) adjusted to operate in a total of 1 × 10^6^ cycles (or until the occurrence of failure in the specimen), in a frequency of 2 Hz, printing a dynamic oblique load (30°) of 130 N ± 10 N on each set [36, 37]. Six specimens were tested at a time, following the same sequence previously established by the randomization process, immersed in distilled water with constant circulation at a temperature of 37°C ± 2°C (Figure 3).

### 3.4. Mechanical Fracture Toughness Test

**Figure 4:**
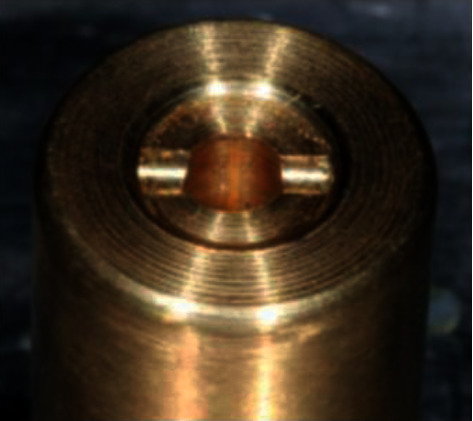
Stainless steel device for positioning the retaining screw.

**Figure 5:**
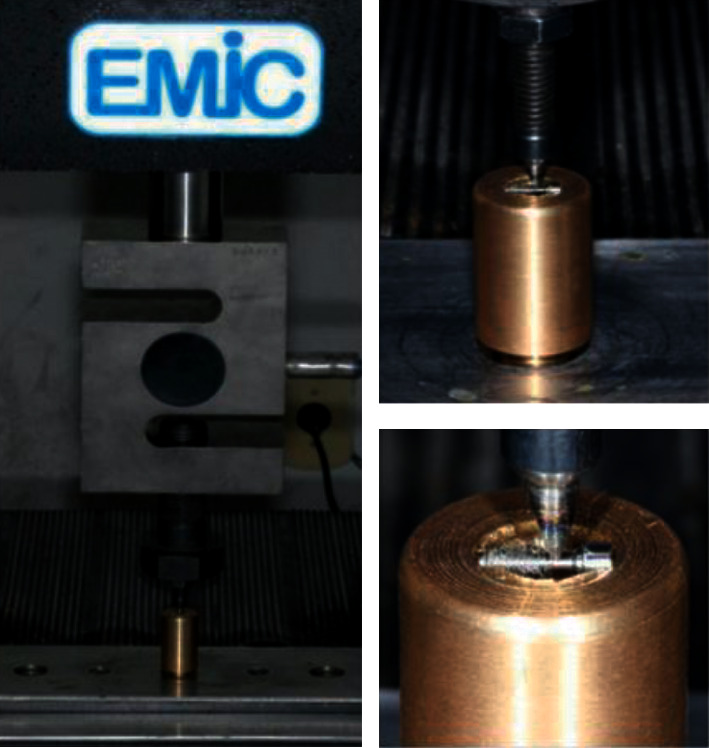
View of the screw positioned in the EMIC with a close-up view of the fracture resistance of the retaining screws.

The fracture strength test was performed by means of the compression test directly on the retention screws of each screw group for each connection type (*n* = 6) after mechanical cycling, analyzing the maximum force (FM). For this, the screws were fixed in a stainless steel device (Figure 4) and positioned in a universal testing machine EMIC®DL-200 (EMIC equipamentos e sistemas de ensaio LTDA, São José dos Pinhais, PR, Brazil) prepared with a load cell of 2000 N and axial displacement speed of 0.5 mm/min [38] (Figure 5). The loading was transmitted to the screws, in its cervical portion, between the smooth surface and the first thread, by means of a chisel-shaped applicator tip until the screw fracture occurred or until the maximum strain force was exceeded and plastic deformation occurred, with decrease of the resistance force, even without the occurrence of fracture.

### 3.5. Statistical Planning

The initial and final torque and destorque data, as well as the fracture strength values obtained were tabulated separately.

Statistical analysis was performed using statistical software (Sigma Plot. 12.1, Systat Software Inc., San José, CA, USA). The data were submitted to the Shapiro–Wilk homogeneity test. For torque maintenance analysis, 3-factor ANOVA was used (factors: alloys, connection, and cycling). The Holm–Sidak test was used with the post hoc technique for multiple comparisons at a 5% significance level. For the fracture strength analysis, a 2-factor ANOVA (factors: alloys and connection) was used, and Tukey's test was used with a post hoc technique for multiple comparisons at a 5% significance level. For interalloy analysis, one-way ANOVA was used, and the Kruskal–Wallis post hoc test for nonparametric data was used for multiple comparisons at a 5% significance level.

## 4. Results

### 4.1. Torque Maintenance

The data obtained regarding the torque maintenance of the retention screws of UCLA abutments, according to the alloys (Ti6Al4V, Ti grade 2, Ti grade 4, Ti grade 4 hard, and surgical steel), the connection pillar/implant (HE and MT), and time (initial period of application of the insertion torque, initial destorque—precycling, and final destorque—postcycling) were transformed in percentage (%).

**Figure 6:**
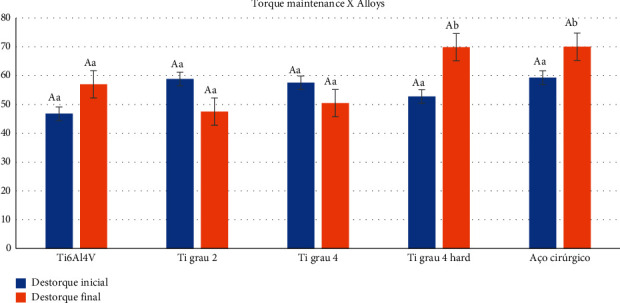
Graphical representation of the means and standard deviation of torque maintenance as a function of the alloys used, independent of the period.

Regarding the alloy factor, regardless of the connections and the period analyzed, the torque loss as a function of the retention screw material did not present a statistically significant difference. When comparing the connection factor independently, in general, it also showed no statistically significant difference, i.e., the connection did not influence the torque values (Table 1 and Figure 6).

**Figure 7:**
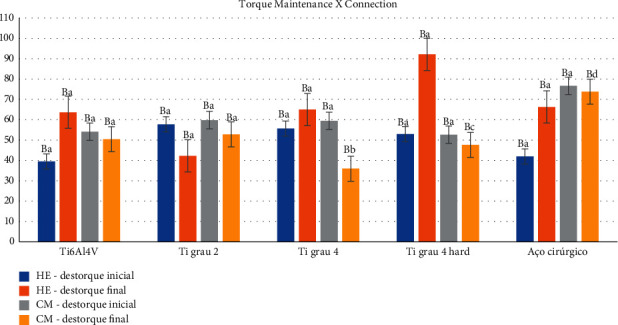
Graphical representation of the means and standard deviations of the torque maintenance considering the HE and MT connections and their respective alloys in the premechanical (initial destorque) and postmechanical cycling (final destorque) periods.

Evaluating the connection factors in relation to the different alloys, regardless of the cycling, in general, there was no statistically significant difference, except for the Ti grade 4 hard (*p*=0.011) for the HE connection, that is, this alloy influenced the value of untorque. Similarly, surgical steel for the MT connection showed a statistically significant difference (*p*=0.017), demonstrating that it also had a positive influence on the destorque value (Table 2 and Figure 7).

Correlating the alloy factors with the connection, in Table 2 and Figure 7, the HE connection did not influence the desorque value, regardless of the alloy type. While for the factors alloys and MT connection, only surgical steel presented a statistically significant difference in relation to the other alloys, showing a mean value of preload maintenance of 73.82% ± 20.69, contrasting with 35.93% ± 18.78 and 47.64% ± 22.83 of the alloys Ti grade 4 (*p*=0.019) and ti grade 4 hard (*p*=0.041), respectively. The other alloys showed no differences among themselves.

Comparing the times used (Table 2 and Figure 7), there was a statistically significant influence on the results of preload maintenance in the initial period of torque application, both in relation to pretorque (*p* < 0.001) and posttorque (*p* < 0.001). Mechanical cycling did not influence the torque values after the confirmation torque was applied, showing no statistically significant difference (*p*=0.220).

**Figure 8:**
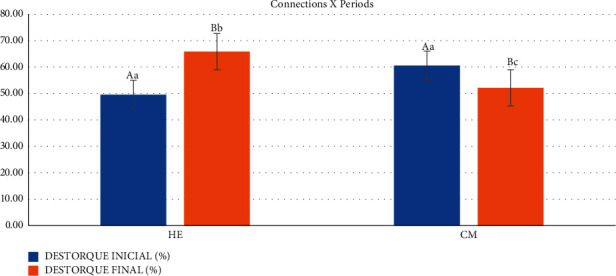
Graphical representation of the averages and standard deviation of the connections in relation to the analyzed untorque periods.

In the comparison between the factors time (precycling and postcycling) and HE connection (Table 3 and Figure 8), there was a significant difference (*p* < 0.001), demonstrating that in this comparison, mechanical cycling had a positive influence on torque maintenance, with an increase of 16.28% compared to the premechanical cycling value. For the MT connection, comparing the initial torque period (20 N) with the initial precycling untorque (60.53% ± 9.64) and final postcycling untorque (52.11% ± 13.75) showed a statistically significant difference (*p* < 0.001), representing the loss of torque inserted into the retention screws.

Finally, comparing connection with time, from confirmation torque, given before mechanical cycling, the connection influenced the maintenance of preload, being better for the HE connection (65.84% ± 17.70) in relation to MT (52.11% ± 13.75), with *p*=0.003 for HE and *p*=0.016 for MT (Table 3 and Figure 8), demonstrating that mechanical cycling negatively influenced the performance of the MT connection.

### 4.2. Fracture Resistance of the Retaining Screws

120 retaining screws of the UCLA abutments were submitted to the fracture strength test by the compression test, 60 for HE connection and other 60 for MT connection, of which 30 of each connection were cycled and other 30 were new, noncycled screws. The means and standard deviations were calculated for maximum force (FM) in kgf (Tables 4 and 5).

**Figure 9:**
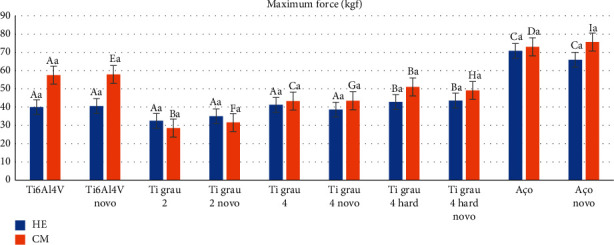
Graphical representation of the means and standard deviations for maximum force (kgf) as a function of alloy type for HE and MT connections.

Analyzing the prosthetic connections according to the alloys (Table 4 and Figure 9), the behavior of the alloys new surgical steel (*p*=0.002) and Ti grade 4 hard (*p*=0.008) influenced the connection performance, showing better results when in the MT connection, with means of 75.68 kgf ± 7.25 and 51.05 kgf ± 5.31. Besides them, Ti6Al4V, regardless of being cycled or new, also showed good results (*p* < 0.001) for the MT connection, with averages 57.54 kgf ± 6.16 and 57.86 kgf ± 3.18, respectively. While the surgical steel cycled screws, new and cycled Ti grade 4, and new and cycled Ti grade 2 screws were not influenced by the connection (*p* > 0.05), they did not present a statistically significant difference.

Correlating the factors alloy and connection, for the HE connection, screws made of surgical steel, regardless of being cycled or not, presented the best performance for this connection, with FM values of 70.82 kgf ± 7.23 and 65.94 kgf ± 9.11, with a statistically significant difference (*p* < 0.005) in relation to all other alloys, except among themselves (*p*=0.849). Besides the steel screws, the new and cycled screws of Ti alloy grade 4 hard (43.59 kgf ± 2.41 and 42.81 kgf ± 1.72, respectively) also showed a statistically significant difference, being superior when compared to the Ti alloy grade 2 screws (32.52 kgf ± 5.49), with *p* < 0.005.

For the MT connection in function of the alloys, the new surgical steel screws with FM of 75.68 kgf ± 7.25 and cycled of 73.05 kgf ± 8.54 also presented the best performance, with a statistically significant difference for all other alloys, *p* < 0.001, except among themselves, *p*=0.997. It was also possible to observe that the cycled screws of Ti6Al4V (57.54 kgf ± 6.16) and new (57.86 kgf ± 3.18), when compared to the screws of Ti grade 2 and Ti grade 4, regardless of being cycled or new, were superior to the other alloys, demonstrating that there is a statistically significant difference in the choice between these alloys (*p* < 0.005), in which the alloy with the worst performance was Ti grade 2. When the screws of these alloys were cycled, they presented an FM value of 28.55 kgf ± 1.44.

**Figure 10:**
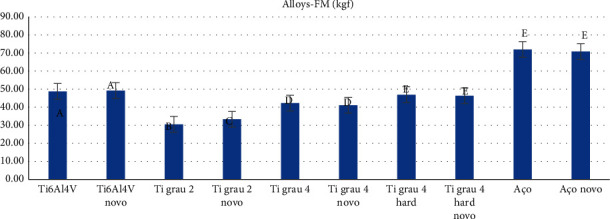
Graphical representation of the means and standard deviations for maximum force (kgf) as a function of the type of metal alloy and its aging (new and cycled), independent of the type of connection.

Finally, when analyzing the FM of different alloy screws, independent of the type of connection, but dependent on aging (new or mechanically cycled screws) (Table 5 and Figure 10), the alloys with the best performances, which showed no statistically significant difference (*p* > 0.005), were surgical steel in cycled screws, surgical steel in new screws, Ti grade 4 hard in cycled and new screws, and Ti6Al4V in new screws.

## 5. Discussion

According to the observed results, the null hypothesis was rejected; on the type of alloy used in the manufacturing of retaining screws, the mechanical cycling and the type of connection influenced the torque maintenance and the fracture resistance of the screws.

Metals can be hot-formed or cold-formed [39]. Whenever they are submitted to plastic deformation, internal defects are generated in their microstructure favoring their mechanical resistance. When the deformation is performed hot, the thermal energy causes the defects created during the process to be eliminated, and the hardening of the metal does not occur. In plastic deformation at low temperatures (cold deformation), the defects generated in the internal microstructure remain stored, consequently increasing the mechanical resistance.

The higher values of destorque (higher preload maintenance) and fracture resistance indicate superiority of surgical steel alloy in relation to Ti alloy grade 4 hard and Ti alloy grade 4, respectively, and especially compared to other alloys.

The use of stainless steels in the manufacture of biomaterials has been widely used until today. Its use is justified mainly by the combination of properties such as good acceptance by the organism, low cost, good formability, high mechanical resistance, and reasonable resistance to corrosion [40, 41]. The higher strength of steel is attributed mainly to the combination of nitrogen and niobium added to its composition during cold forming. These additions promote the hardening of its particles during the steel's recrystallization process [42]. Moreover, nitrogen favors its process during plastic deformation, which ensures its good mechanical resistance [43].

Regarding the metallic alloys, the Ti alloy grade 4 hard showed influence on the destorque value in this study, presenting good results for the retaining screws. This result corroborates the study by Elias and coworkers [44] who after mechanical evaluations of Ti alloys grade 2, Ti6Al4V, Ti grade 4, and grade 4 hard involving tension, compression, hardness, and torque tests, the Ti alloy grade 4 hard showed superior mechanical strength, as well as in the present study, revealing an improvement in the mechanical properties of this material. Another result published by the same authors, which is in agreement with the present study, is that when comparing the alloys in the compression test, Ti grade 4 hard presented the highest result when compared to Ti grades 2 and 4, remaining close to Ti6Al4V.

In plastic deformation at low temperatures (cold), the defects generated in the internal microstructure remain stored, consequently increasing the mechanical strength. This phenomenon is called strain hardening. From this mechanism can be obtained the hardened Ti grade 4, also called Ti cp4 hard, whose chemical composition is identical to Ti grade 4 [39, 44]. This alloy, recently used for dental implants, has shown higher mechanical strength than the other alloys (Ti cp and Ti6Al4V) [45].

Although Ti6Al4V showed satisfactory results in this study [46], two clinical cases of reactive lesions in the periimplant mucosa were reported, which were diagnosed as pyogenic granuloma and peripheral giant cell granuloma. In histopathological analysis, they observed vascular proliferation, intense inflammatory infiltrate, and metal-like particles. They suggested that these fragments were Ti ions released into the periimplant mucosa due to the corrosive process of the implant's prosthetic platform surface [46, 47]. Among the ions released by Ti6Al4V alloy, vanadium (V) is considered a highly cytotoxic metal and a cause of foreign body reaction [48], and aluminum (Al) is related to neurological disorders, such as Alzheimer's disease [49]. Thus, eventhough its mechanical properties are excellent, its biocompatibility is under suspicion, and its use is not the first-choice indication for implants and their prosthetic components.

When analyzing the type of prosthetic connection with mechanical cycling, according to some studies [8, 50], the Morse taper was considered a mechanically superior connection to the external hexagon. However, in this study, the external hexagon was the one that showed better postcycling torque maintenance, unlike the Morse taper, which showed a statistically significant negative difference between precycling (after confirmation torque) and postmechanical cycling (final torque) periods. Corroborating with this research, some studies have also demonstrated that HE showed better torque maintenance compared to retention screws [51, 52], and for Kim and collaborators [52], the loss of preload, after mechanical cycling, depends specifically on the type of abutment and the characteristics of the abutment/implant connection design, besides the wider implant diameter being more advantageous in relation to torque loss [17].

In the literature review [53], even with the consensus established by several authors that the internal connection associated with the MT is the most fatigue-resistant type of connection, screw loosening is considered a multifactorial event that depends not only on the type of connection but also on the design and material of making the retention screw, type and design of the prosthetic abutments, direction of occlusal forces, and premature contact points among other factors.

For other authors [54], untorque values close to or greater than the applied insertion torque indicate a good prognosis for the connections in question, as occurred with the external hexagon connection in this study. The superiority of the hexagon may also mean that this connection has undergone mechanical transformations, conferring improvements in the material and, consequently, demonstrating good performance, related to the type of raw material and manufacturing quality, which differ between manufacturers.

As shown in this study, the lower torque maintenance for the Morse taper, which has an internal connection, is similar to the research of Lee and colleagues [55] and others [17, 55], when they stated that in the external hexagonal implant system, because they have greater thickness in their lateral walls, they are more resistant to compressive forces during the reception of occlusal loads, leading to a lower axial displacement of the abutment on the implant [56, 57]. This displacement can cause biomechanical complications such as loosening of the retention screw, leading to prosthetic instability and maladjustment, which justifies the lower torque maintenance in Morse tapers observed in this study.

In this study, the confirmation torque influenced the torque values. This fact can also be observed in other studies [58, 59], which showed a progressive decrease in the torque value. In this study, it was 55.04%, regardless of the connection, after application of the insertion torque. When the initial torque is applied, it is lost even when the screwed joint is not subjected to the application of any external force [60]. This can be explained by the accommodation or inclusion relaxation of the retaining screw in the abutment/implant connection, called the sedimentation effect [61]. To prevent this decrease in insertion torque, some manufacturers recommend retightening the screw after 10 minutes [62]. In this study, the confirming torque was applied 3 minutes after the initial torque, with satisfactory results.

In the fracture resistance test, 60 new screws of the two connections were submitted, in addition to the screws that had already been submitted to mechanical cycling. In general, the new screws had the best results of maximum strength, which indicates that they resisted more to the forces on them before failure, which may indicate that mechanical cycling changes the capacity of the retention screws to resist to external forces and may lead to their fracture in the long term.

When comparing the prosthetic connections, the screws from the Morse taper connection showed the highest means of maximum force, although very close to the HE means. For this connection, the new screws were also superior to the cycled ones, but with very close values, demonstrating their biomechanical stability.

Regarding the metallic alloys used during the compression test, the ones that showed better mechanical performance were the surgical steel alloy, followed by the alloy Ti grade 4 hard and Ti grade 4. Surgical steel, despite its high strength, still presents corrosive characteristics [41, 45]. The hard Ti grade 4 alloy, for Elias CN and collaborators, after being submitted to severe plastic deformation, became more resistant to compression than the conventionally made alloy [24]. The worst performance was demonstrated by the Ti grade 2 alloy, which, as previously mentioned, presents low potential mechanical strength and low wear resistance, inhibiting its use for biomedical applications [24], in addition to improving its mechanical properties hampered by the possible reduction of its biocompatibility [21].

The relevance of this study lies in the fact that it demonstrates clinical situations, in which this “in vitro” research simulated occlusal forces for the stability of the screwed connection with retention screws of different metallic alloys of UCLA abutments, in which the screws were submitted to the torque recommended by the manufacturer. Since the torque applied tends to decrease with time, it is essential to perform periodic evaluations to maintain an adequate torque, thus avoiding loosening and, consequently, fracture of the retention screws of the screwed junctions.

The range of potential sources of bias as this is an in vitro assay limits the full representation of the results as it does not show the response of a living being/tissue to a material/drug; it is merely a control and a baseline for the next stage of development. So, more needed is long-term randomized clinical trials to establish a real approach to this approach for future clinical practice.

## 6. Conclusion

The different associated materials, a mechanical cycling, and the type of connection did not influence the maintenance of torque and fracture resistance of the retention screws in different exclusives.

Based on the results obtained and within the methodological limitations of this in vitro study, we can conclude the following:

- For the MT connection, the alloys that showed the best performance in maintaining torque were surgical steel, Ti grade 4 hard, and Ti grade 4
- Mechanical cycling negatively influenced torque maintenance for this connection
- The compression test showed that new screws have higher fracture resistance, suggesting the periodic replacement of the retaining screws in use
- The metallic alloys influenced the mechanical resistance of the retention screws, being the surgical steel the most resistant and the Ti grade 2 alloy the least resistant, differently from the torque maintenance, in which the alloys, when analyzed independently, presented similar behavior

## Figures and Tables

**Table 1:** Means and standard deviation of torque maintenance (%) as a function of alloys used, independent of the period.

Alloys	Destorque initial	Destorque final
Ti6Al4V	46.79 ± 10.33^Aa^	57.01 ± 9.35^Aa^
Ti grau 2	58.83 ± 1.50^Aa^	47.50 ± 7.43^Aa^
Ti grau 4	57.54 ± 2.65^Aa^	50.45 ± 20.53^Aa^
Ti grau 4 hard	52.79 ± 0.23^Aa^	69.89 ± 31.46^Ab^
Surgical steel	59.28 ± 24.53^Aa^	70.03 ± 5.35^Ab^

^*∗*^Distinguished capital letters represent a statistically significant difference in the rows. Distinguished lower case letters represent a statistically significant difference in the columns. Source, prepared by the author.

**Table 2:** Means and standard deviation of torque maintenance (destorque) considering HE and MT connections and their respective alloys in the periods premechanical (initial destorque) and postmechanical cycling (final destorque).

Connection	Alloys	Torque initial (N)	Destorque initial (%)	Destorque final (%)
HE	Ti6Al4V	30^Aa^	39.49 ± 20.82^Ba^	63.63 ± 18.11^Ba^
	Ti grau 2	30^Aa^	57.76 ± 11.74^Ba^	42.24 ± 20.21^Ba^
	Ti grau 4	30^Aa^	55.66 ± 23.83^Ba^	64.97 ± 25.91^Ba^
	Ti grau 4 hard	30^Aa^	52.96 ± 33.44^Ba^	92.13 ± 27.87^Ba^
	Surgical steel	30^Aa^	41.93 ± 18.07^Ba^	66.25 ± 26.99^Ba^
MT	Ti6Al4V	20^Aa^	54.10 ± 26.15^Ba^	50.39 ± 22.25^Ba^
	Ti grau 2	20^Aa^	59.89 ± 18.47^Ba^	52.76 ± 18.13^Ba^
	Ti grau 4	20^Aa^	59.42 ± 17.04^Ba^	35.93 ± 18.78^Bb^
	Ti grau 4 hard	20^Aa^	52.63 ± 12.74^Ba^	47.64 ± 22.83^Bc^
	Surgical steel	20^Aa^	76.63 ± 7.74^Ba^	73.82 ± 20.69^Bd^

^*∗*^Distinguished capital letters represent a statistically significant difference in the rows. Distinguished lower case letters represent a statistically significant difference in the columns. Source, prepared by the author.

**Table 3:** Means and standard deviation of the distortions as a function of different connections and periods of distortion (precycling and postcycling), independent of the screw alloys.

Connection	External hexagon	Cone morse
Destorque initial	49.56 ± 8.29^Aa^	60.53 ± 9.54^Aa^
Destorque final	65.84 ± 17.70^Bb^	52.11 ± 13.75^Bc^

^*∗*^Distinguished capital letters represent a statistically significant difference in the rows. Distinguished lower case letters represent a statistically significant difference in the columns. Source, prepared by the author.

**Table 4:** Means and standard deviations for maximum force (kgf) as a function of alloy type for HE and MT fittings.

Connections	Alloys	Maximum force (kgf)
HE	Ti6Al4V	40.01 ± 1.22^Aa^
	Ti grau 2	32.52 ± 5.49^Aa^
	Ti grau 4	41.25 ± 2.96^Aa^
	Ti grau 4 hard	42.81 ± 1.72^Ba^
	Surgical steel	70.82 ± 7.23^Ca^
	Ti6Al4V novo	40.59 ± 1.60^Aa^
	Ti grau 2 novo	35.01 ± 6.24^Aa^
	Ti grau 4 novo	38.66 ± 1.78^Aa^
	Ti grau 4 hard novo	43.59 ± 2.41^Ba^
	New surgical steel	65.94 ± 9.11^Ca^
MT	Ti6Al4V	57.54 ± 6.16^Aa^
	Ti grau 2	28.55 ± 1.44^Ba^
	Ti grau 4	43.27 ± 1.65^Ca^
	Ti grau 4 hard	51.05 ± 5.31^Ba^
	Surgical steel	73.05 ± 8.54^Da^
	Ti6Al4V novo	57.86 ± 3.18^Ea^
	Ti grau 2 novo	31.58 ± 6.68^Fa^
	Ti grau 4 novo	43.53 ± 8.30^Ga^
	Ti grau 4 hard novo	49.12 ± 3.62^Ha^
	New surgical steel	75.68 ± 7.25^Ia^

^*∗*^Distinguished capital letters in the rows indicate a statistically significant difference. Distinguished lower case letters in the columns represent a statistically significant difference. Source, prepared by the author.

**Table 5:** Means and standard deviations for maximum force (kgf) as a function of alloy type and aging (new and cycled), independent of connection type.

Alloys	Maximum force (kgf)
Ti6Al4V cycled	48.77 ± 12.40^A^
Ti6Al4V new	49.23 ± 12.21^A^
Ti grau 2 cycled	30.53 ± 2.80^B^
Ti grau 2 new	33.29 ± 2.42^C^
Ti grau 4 cycled	42.26 ± 1.42^D^
Ti grau 4 new	41.09 ± 3.44^D^
Ti grau 4 hard cycled	46.93 ± 5.82^E^
Ti grau 4 hard new	46.35 ± 3.90^E^
Cycled surgical steel	71.93 ± 1.57^E^
New surgical steel	70.81 ± 6.89^E^

^*∗*^Distinct capital letters in the column represent statistically significant difference. Source, prepared by the author.

## Data Availability

The data that support the findings of this study are available in the supplementary material of this article.
